# Highly Pathogenic Avian Influenza Virus Subtype 5 in Cetaceans, Brazil

**DOI:** 10.3201/eid3210.260051

**Published:** 2026-10

**Authors:** Alanis S. Melgarejo, Mauricio Tavares, Derek B. de Amorim, Sergio C. Estima, Andrine Paiva, Caio R. Soares-Oliveira, Liane A. Dias, Renan C. de Lima, Silvina Botta, Eduardo R. Secchi, Barbara A. Davila, Carlos de Oliveira, Larissa S. Heinzelmann, Martha T. Oliveira, Vitor L. Carvalho, Aline R. Souza, Salvatore Siciliano, Paula Baldassin, Greicy F. Ruenes, Paulo H. Ott, Luciana Sonne, Ana C. Franco, Larissa R. Oliveira

**Affiliations:** Universidade Federal do Rio Grande do Sul, Porto Alegre, Brazil (A.S. Melgarejo, M. Tavares, D.B. de Amorim, M.T. Oliveira, L. Sonne, A.C. Franco); Núcleo de Educação e Monitoramento Ambiental, Rio Grande, Brazil (S. C. Estima, A. Paiva, C.R. Soares-Oliveira); Universidade Federal do Rio Grande, Rio Grande (L.A. Dias, R.C. de Lima, S. Botta, E.R. Secchi); Universidade do Vale do Rio dos Sinos, São Leopoldo, Brazil (B.A. Davila, C. de Oliveira, L.S. Heinzelmann); Associação de Pesquisa e Preservação de Ecossistemas Aquáticos, Caucaia, Brazil (V.L. Carvalho, A.R. Souza, L.R. Oliveira); Fundação Oswaldo Cruz, Escola Nacional de Saúde Pública, Rio de Janeiro, Brazil (S. Siciliano); Grupo de Estudos da Região dos Lagos, Rio de Janeiro (S. Siciliano, G.F. Ruenes); Instituto BW para a Conservação e Medicina da Fauna Marinha, Araruama, Brazil (P. Baldassin); Universidade Estadual do Rio Grande do Sul, Osório, Brazil (P.H. Ott); Grupo de Estudos de Mamíferos Aquáticos do Rio Grande do Sul, Porto Alegre, Brazil (P.H. Ott, L.R. Oliveira); Marine Vertebrate Evolution and Conservation Lab, Fortaleza, Brazil (L.R. Oliveira).

**Keywords:** influenza, HPAIV, wildlife disease surveillance, emerging viral diseases, One Health, cetaceans, virus, panzootic, zoonoses, H5N1, Brazil

## Abstract

We identified highly pathogenic avian influenza virus subtype H5 in stranded dolphins along the coastline of Brazil during 2023–2025. Infected animals included species classified as vulnerable or endangered. Our results highlight the need for ongoing surveillance of cetaceans susceptible to viral infections, which pose an additional threat to threatened species.

Marine ecosystems in Brazil have high biodiversity and support numerous top predators, including marine mammals. Along the coast of Brazil, >46 cetacean species have been recorded, several of which are classified as threatened or near threatened (*1*). 

Influenza A viruses (IAV) infect a wide range of hosts, including marine mammals (*2*). The highly pathogenic avian influenza (HPAI) H5N1 virus is causing a widespread panzootic, including outbreaks with high mortality rates among birds and pinnipeds (*3*). Reports of IAV in cetaceans remain rare and limited to a few species, and large-scale outbreaks have not been reported (*4*). We screened swab samples from cetaceans stranded along the coast of Brazil during November 2023–August 2025 for IAV by using quantitative reverse transcription PCR (qRT-PCR).

During November 2023–August 2025, we recorded stranded marine mammals along the Brazil coastlines of Rio Grande do Sul (≈485 km), Rio de Janeiro (317 km), and Ceará (573 km) (Figure 1, panels A–D). Moreover, we conducted systematic surveys along the beaches of Rio Grande do Sul and responded to stranding notifications across all coastal regions. For each animal, we recorded standardized data including date, location, species, sex, and decomposition stage (*5*). Ethical approval for this study was granted by the Animal Ethics Committee of the Universidade do Vale do Rio dos Sinos (protocol no. PPECEUA 02.2025).

**Figure 1:**
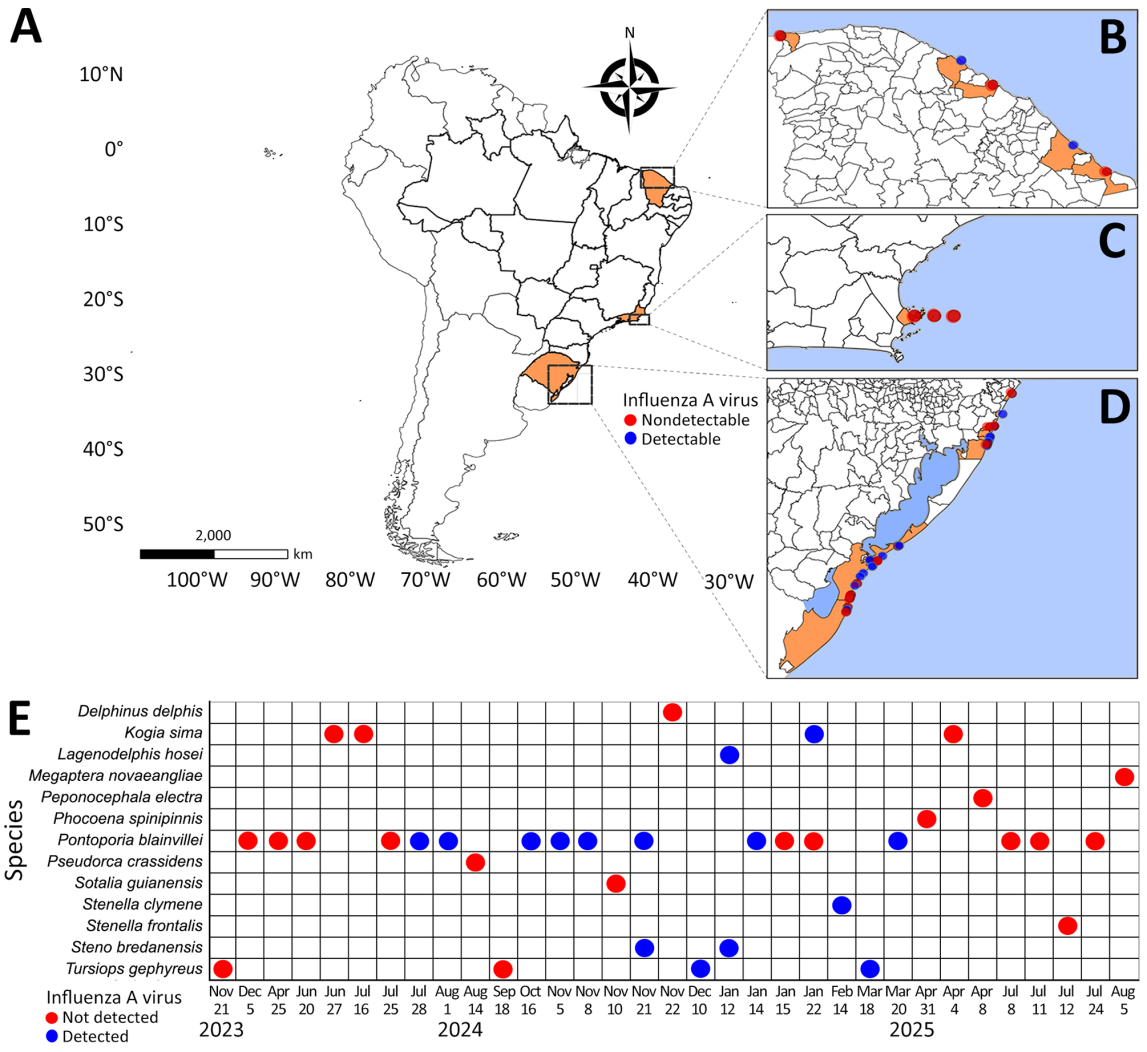
Temporal and spatial distribution of cetacean species collected along the coast of Brazil during a study of influenza A virus from November 2023–August 2025. A–D) Sampling locations along the coast of Brazil. Insets show sampling locations in the states of Ceará (B), Rio de Janeiro (C), and Rio Grande do Sul (D). Each dot represents an analyzed sample from the cetacean. E) Temporal distribution of sampled cetaceans. Each point represents an individual animal; red points indicate detection of influenza A virus matrix gene RNA, and blue points indicate no detectable viral RNA.

We collected swab samples exclusively from carcasses in decomposition stages 2–4, which include the bloated stage, active decay, and advanced decay (*5*). We collected and fixed tissue samples (brain, lungs, trachea, spleen, intestine and liver) from carcasses in earlier decomposition stages (*2*,*3*) in 10% buffered formalin and routinely processed the fixed tissues for histology. We extracted total nucleic acids by using the MagMAX CORE Nucleic Acid Purification Kit (Thermo Fisher Scientific, https://www.thermofisher.com) with the KingFisher Flex system (Thermo Fisher Scientific). We performed qRT-PCR amplification of the IAV matrix gene for IAV detection (*6*), and we detected the H5N1 strain by using World Health Organization molecular protocols (*7*). We set the fluorescence threshold at 0.09 and classified samples with cycle threshold (Ct) values <37 as positive. We evaluated the sensitivity of the IAV qRT-PCR by using RNA from MDCK cells infected with H1N1, cloned into a plasmid as a positive control. We assessed the sensitivity of the H5N1 qRT-PCR assay by using a pEz plasmid and performed 10-fold serial dilutions to determine the qRT-PCR limit of detection.

We analyzed 45 stranded cetaceans, representing 13 species (Figure 1, panel E; Appendix Table 1). We detected the IAV matrix gene RNA in 18 animals, including Franciscana dolphins (*Pontoporia blainvillei*), Lahille’s bottlenose dolphins (*Tursiops truncatus gephyreus*), rough-toothed dolphins (*Steno bredanensis*), Clymene dolphins (*Stenella clymene*), dwarf sperm whales (*Kogia sima*), and Fraser’s dolphins (*Lagenodelphis hosei*). Samples positive for IAV RNA were from multiple tissues, most frequently blowhole swab samples, followed by cerebral, oral, anal, intestinal, tracheal, and ocular tissue samples. Overall, viral RNA levels were low, and Ct values ranged from 28–37, corresponding to ≈1.3 × 10^5^ –8.0 × 10^2^ RNA copies/µL (Figure 2, panel A), approaching the assay limit of detection (200–2,000 RNA copies/µL). We only detected the H5N1 strain in 2 samples, both with viral RNA levels near the assay limit of detection (Figure 2, panel B; Appendix Table 2). Our histopathologic examination of the 6 samples positive for IAV (animals 11, 16, 17, 35, 37 and 39; Appendix Tables 1, 2) did not reveal lesions associated with influenza infection.

**Figure 2:**
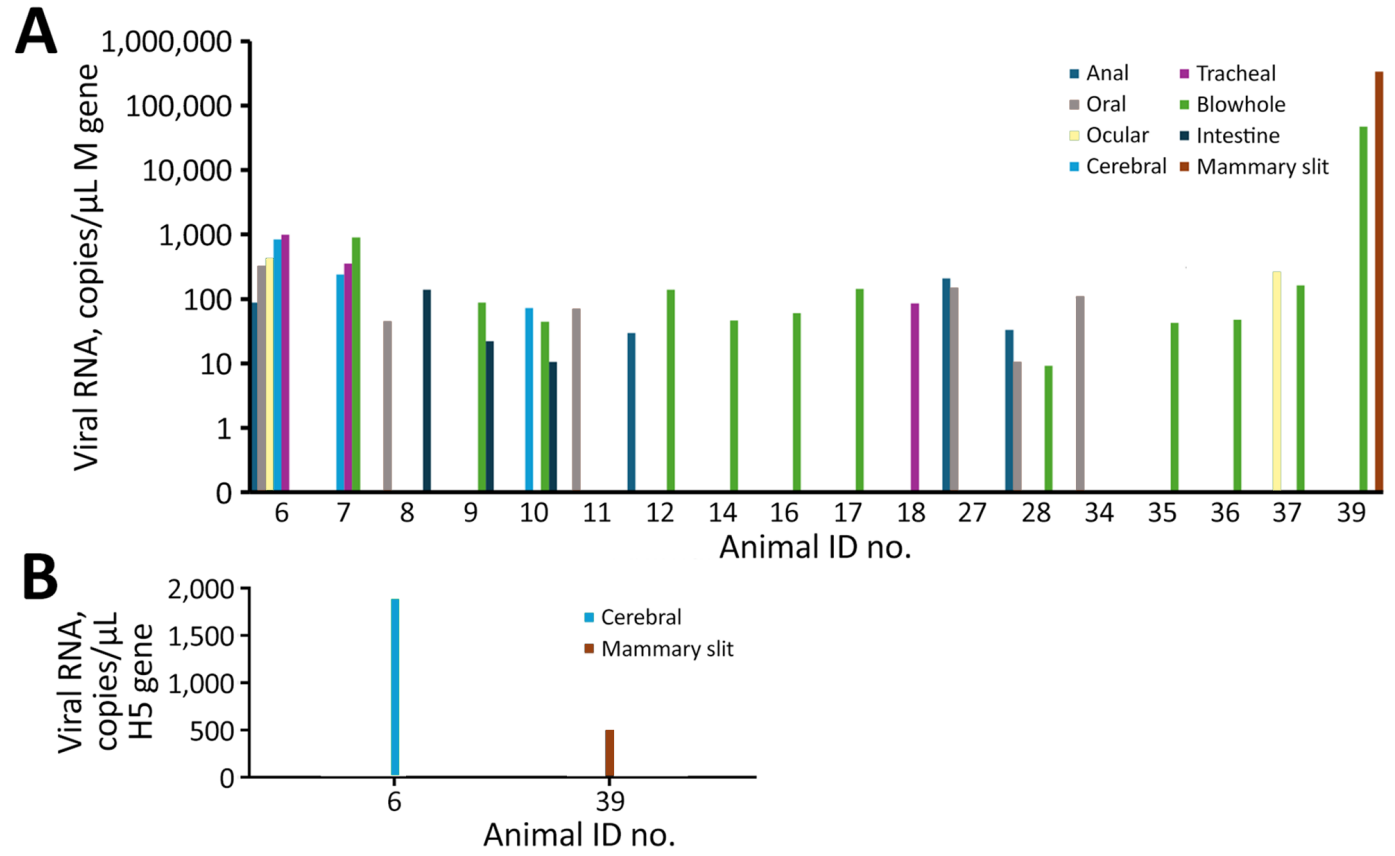
Influenza A viral RNA detected by quantitative reverse transcription PCR in stranded cetaceans from Brazil, November 2023–August 2025. A) Influenza A virus M gene RNA concentration (copies/µL) in positive animals by sample type. B) H5 subtype RNA concentrations (copies/µL) in cetaceans positive for H5 influenza A virus by sample type. ID, identification; M, matrix.

We detected IAV in samples from cetaceans along the coastline of Brazil after large HPAI H5N1 outbreaks that caused high mortality rates in pinnipeds along the coastline of South American were reported in 2023, which suggests enduring viral circulation in the marine environment and interspecies transmission (*3*,*9*). Cetaceans with samples positive for IAV included the Franciscana dolphin, which is classified as vulnerable, and Lahille’s bottlenose dolphin, which is classified as endangered (*8*). Our results underscore a new potential threat to species already at risk for extinction along the coast of Brazil, particularly for Franciscana and Lahille’s bottlenose dolphins, native to the southwestern Atlantic Ocean. Moreover, we detected the HPAI H5N1 strain in Franciscana and Clymene dolphins, highlighting the potential for viral dissemination in coastal and offshore waters.

We observed high Ct values, corresponding with low viral RNA copy numbers. Those results likely reflect RNA degradation associated with postmortem autolysis and advanced carcass decomposition, which can reduce RNA integrity and limit viral detection (*10*). Indeed, the likelihood of identifying lesions associated with influenza infection was likely reduced by the limited number of animals available for histopathologic evaluation because of the advanced stage of decomposition in most of the carcasses analyzed. Environmental contamination from seabirds and shorebirds cannot be excluded in stranded carcasses. However, we detected viral RNA in internal tissues, which indicates true infection rather than surface contamination. Our results underscore the importance of continued and systematic surveillance of marine mammals to understand pathogen dynamics at the wildlife–marine interface.

Appendix Additional information about highly pathogenic avian influenza virus subtype 5 in cetaceans, Brazil.
