# Supplementary material for: Highly Pathogenic Avian Influenza Virus Subtype 5 in Cetaceans, Brazil
Source: Emerg Infect Dis. 2026 Oct;32(10):1746–9. doi: 10.3201/eid3210.260051 (PMC13632873; doi:10.3201/eid3210.260051)
Supplement: Supplementary file 1 [file 26-0051-Techapp-s1.pdf]

Article DOI: https://doi.org/10.3201/eid3108.260051

EID cannot ensure accessibility for supplementary materials supplied by authors. Readers who have difficulty accessing supplementary content should contact the authors for assistance.

Highly Pathogenic Avian Influenza Virus Subtype 5 in Cetaceans, Brazil

Appendix

Appendix Table 1. Overview of Sample Collection. This table presents the details of the samples collected.

Code number	Species	Gender	CT (cm)	Geraci	Code sample	Site collection	Latitude	Longitude	Collection date	Samples
1	*Pontoporia blainvillei*	M	ND	4	Pb #6	Rio Grande	32° 44′ 57″ S	52° 27′ 9″ W	2023–12–05	Blowhole
2	*Pontoporia blainvillei*	F	114	3	MM297	Tramandaí (RS)	30° 0′ 23,5″ S	50° 11′ 53″ W	2024–04–25	Cerebral<br>Right Cerebellum<br>Left Cerebellum<br>Blowhole<br>Tracheal<br>Anal<br>Oral<br>Feces<br>Ocular<br>Genital
3	*Pontoporia blainvillei*	M	96	3	MM288	Cidreira (RS)	30° 10′ 0,9″ S	50° 11′ 53″ W	2024–04–25	Cerebral<br>Tracheal<br>Oral<br>Anal<br>Blowhole<br>Ocular<br>Genital
4	*Pontoporia blainvillei*	F	120, 5	3	MM301	Arroio do Sal (RS)	29° 30′ 12,8″ S	49° 50′ 59″ W	2024–06–20	Blowhole<br>Anal<br>Ocular<br>Oral<br>Cerebral<br>Tracheal
5	*Pontoporia blainvillei*	F	140	2	PB#1	Rio Grande (RS)	31° 49′ 21″ S	51° 39′ 16″ W	2024–07–16	Anal<br>Oral
6	*Pontoporia blainvillei*	M	112	2	MM307	Cidreira (RS)	30° 11′ 33″ S	50° 12′ 30″ W	2024–07–23	Anal<br>Blowhole<br>Oral<br>Ocular<br>Cerebral<br>Tracheal
7	*Pontoporia blainvillei*	M	104, 2	3	MM309	Tramandaí (RS)	30° 0′ 20″ S	50° 7′ 54″ W	2024–08–01	Anal<br>Blowhole<br>Oral<br>Ocular<br>Genital<br>Cerebral<br>Tracheal
8	*Pontoporia blainvillei*	F	143	4	MM315	Cidreira (RS)	30° 9′ 34″ S	50° 11′ 44″ W	2024–10–16	Blowhole<br>Anal<br>Oral<br>Genital<br>Small intestine
9	*Pontoporia blainvillei*	M	ND	4	MM316	Balneário Pinhal (RS)	30° 14′ 12″ S	50° 13′ 31″ W	2024–10–16	Tracheal<br>Blowhole<br>Small intestine
10	*Pontoporia blainvillei*	U	137	4	MM317	Balneário Pinhal (RS)	30° 17′ 50″ S	50° 14′ 58″ W 5	2024–10–16	Blowhole<br>Cerebral<br>Small intestine<br>Large intestine
11		M	135	3	P. b 1					Blowhole

Code number	Species	Gender	CT (cm)	Geraci	Code sample	Site collection	Latitude	Longitude	Collection date	Samples
	Pontoporia blainvillei					Rio Grande (RS)	31° 48′ 54″ S	51° 38′ 17″ W	2024–11–05	Anal<br>Oral
12	*Pontoporia blainvillei*	M	142	4	P. b 1	Rio Grande (RS)	32° 13′ 59″ S	52° 12′ 22″ W	2024–11–08	Blowhole
13	*Pontoporia blainvillei*	M	106	3	P.b 3	Rio Grande (RS)	32° 16′ 57″ S	52° 15′ 12″ W	2024–11–08	Blowhole<br>Anal<br>Tracheal<br>Cerebral
14	*Pontoporia blainvillei*	F	150	I	P. b 2	Rio Grande (RS)	ND	ND	2024–11–21	Blowhole<br>Oral
15	*Pontoporia blainvillei*	U	138	2	P. b 1	Rio Grande (RS)	31° 58′ 20″ S	51° 54′ 9″ W	2025–01–14	Blowhole<br>Oral
16	*Pontoporia blainvillei*	M	92	3	P. b 2	Rio Grande (RS)	32° 7′ 44″ S	52° 3′ 48″ W	2025–01–14	Blowhole<br>Anal<br>Oral
17	*Pontoporia blainvillei*	F	109	3	P. b 3	Rio Grande (RS)	32° 2′ 48″ S	51° 59′ 0″ W	2025–01–14	Blowhole<br>Anal<br>Oral<br>Tracheal
18	*Pontoporia blainvillei*	U	107	2	P. b 2	Rio Grande (RS)	32° 37′ 6″ S	52° 25′ 13″ W	2025–01–15	Blowhole<br>Oral
19	*Pontoporia blainvillei*	M	128	2	P. b 1	Rio Grande (RS)	32° 23′ 26″ S	52° 18′ 11″ W	2025–01–22	Blowhole<br>Oral
20	*Pontoporia blainvillei*	M	123	2	P. b 2	Rio Grande (RS)	32° 47′ 11″ S	52° 27′ 49″ W	2025–03–20	Blowhole<br>Anal<br>Oral<br>Tracheal
21	*Pontoporia blainvillei*	M	108, 5	3	MM330	Tramandaí (RS)	30° 5′ 30″ S	50° 10′ 10″ W	2025–07–09	Cerebral<br>Tracheal<br>Intestine
22	*Pontoporia blainvillei*	F	140	2	Pb 12/07/24	Rio Grande (RS)	32° 49′ 12″ S	52° 28′ 35″ W	2025–07–12	Nasal<br>Anal<br>Oral<br>Tracheal
23	*Pontoporia blainvillei*	F	105	2	MM331	Cidreira (RS)	30° 8′ 30″ S	50° 11′ 19″ W	2025–07–24	Oral<br>Blowhole<br>Tracheal<br>Lung<br>Ocular<br>Cerebral<br>Anal
24	*Pontoporia blainvillei*	F	142	2	MM333	Balneário Pinhal (RS)	30° 16′ 19″ S	50° 14′ 23″ W	2025–07–24	Oral<br>Nasal<br>Tracheal<br>Ocular<br>Lung<br>Cerebral
25	*Tursiops gephyreus*	M	132	4	Tg#1	Rio Grande (RS)	32° 2′ 33″ S	51° 58′ 48″ W	2023–11–21	Blowhole
26	*Tursiops gephyreus*	M	169	3	Tg#1	Rio Grande (RS)	32° 7′ 24″ S	52° 3′ 30″ W	2024–09–18	Anal
27	*Tursiops gephyreus*	M	278	4	T. g 1	Rio Grande (RS)	ND	ND	2024–12–10	Blowhole<br>Anal<br>Oral
28	*Tursiops gephyreus*	M	326	3	T. g 1	Rio Grande (RS)	32° 7′ 41″ S	52° 3′ 47″ W	2025–02–18	Blowhole<br>Anal<br>Oral
29	*Delphinus delphis*	M	130	2	D.d 1	Rio Grande (RS)	32° 33′ 40″ S	52° 24′ 1″ W	2024–06–27	Blowhole<br>Anal
30	*Delphinus delphis*	F	208	ND			ND	ND	2024–07–28	Blowhole<br>Anal

Code number	Species	Gender	CT (cm)	Geraci	Code sample	Site collection	Latitude	Longitude	Collection date	Samples
					D. delphis 1	Rio Grande (RS)				Ocular<br>Traqueal
31	*Delphinus delphis*	F	203	2	D.d 1	Rio Grande (RS)	ND	ND	2024–11–22	Blowhole<br>Anal<br>Oral<br>Ocular
32	*Pseudorca crassidens*	M	350	2	Pc#1	Rio Grande (RS)	32° 35′ 22″ S	52° 24′ 39″ W	2024–08–14	Blowhole<br>Anal<br>Ocular<br>Oral
33	*Sotalia guinensis*	M	ND	3	S.g 10/11/24	São Gonçalo do Amarante (CE)	3° 31′ 28″ S	38° 52′ 17″ W	2024–11–10	Edema
34	*Steno bredanensis*	M	269	2	S.b 3	Rio Grande (RS)	32° 37′ 8″ S	52° 25′ 13″ W	2024–11–21	Blowhole<br>Oral<br>Anal
35	*Steno bredanensis*	M	ND	3	S.b 12/01/25	Beberibe (CE)	4° 17′ 17″ S	37° 57′ 54″ W	2025–01–12	Blowhole<br>Genital
36	*Lagenodelphis hosei*	M	ND	3	L.h 12/01/25	Trairi (CE)	3° 13′ 22″ S	39° 15′ 7″ W	2025–01–12	Blowhole
37	*Kogia sima*	F	223	2	K.s 2	Rio Grande (RS)	32° 25′ 10″ S	52° 20′ 18″ W	2025–01–22	Blowhole<br>Anal<br>Oral<br>Ocular
38	*Kogia sima*	M	ND	2	K.s 04/04/25	Aracati (CE)	4° 37′ 39″ S	37° 35′ 15″ W	2025–04–04	Blowhole<br>Anal
39	*Stenella clymene*	F	192	2	MM324	Xangri-lá (RS)	29° 49′ 1″ S	50° 2′ 31″ W	2025–02–14	Ocular<br>Tracheal<br>Mammary slit<br>Vaginal<br>Blowhole<br>Oral<br>Anal<br>Cerebral
40	*Phocoena spinipinnis*	U	164	4	MM325	Tramandaí (RS)	−29,997356	−50,128046	2025–03–31	Tracheal<br>Lungs<br>Cerebral<br>Intestine
41	*Peponocephala electra*	F	ND	4	P.e 08/04/25	Barroquinha (CE)	2° 54′ 25″ S	41° 17′ 38″ W	2025–04–08	Blowhole<br>Tracheal
42	*Stenella frontalis*	M	ND	2	354375	Armação dos Búzios (RS)	22° 46′ 17″ S	41° 54′ 57″ W	2025–07–12	Lung<br>Anal<br>Ocular<br>Tracheal<br>Cerebral
43	*Stenella frontalis*	F	ND	2	354479	Armação dos Búzios (RS)	22° 46′ 17″ S	41° 54′ 57″ W	2025–07–12	Lung<br>Anal<br>Ocular<br>Tracheal<br>Cerebral
44	*Stenella frontalis*	F	ND	2	354444	Armação dos Búzios (RS)	22° 46′ 17″ S	41° 54′ 57″ W	2025–07–12	Lung<br>Anal<br>Ocular<br>Tracheal<br>Cerebral
45	*Megaptera novaeangliae*	F	880	2	MM335	Arroio do Sal (RS)	29° 31′ 54″ S	49° 52′ 13″ W	2025–08–09	Lung<br>Cerebral<br>Ocular<br>Oral<br>Blowhole<br>Anal

Abbreviations: U, undetermined; ND, not determined.

Appendix Table 2. Overview of positive samples. Threshold cycle (Ct) values obtained by RT-qPCR for FLUAV(M gene) and H5 (clades 1, 2, and 3), along with corresponding viral RNA (copies/μL).

Code number	Species	Samples	Ct influenza A (M gene)	Viral RNA (copies/ μL) M gene	Ct H5 (clade 1,2,3)	Viral RNA (copies/ μL) M gene
6	*Pontoporia blainvillei*	Anal	34,14	875,75	ND	ND
		Oral	32,32	3254,5	ND	ND
		Ocular	31,84	4313,5	ND	ND
		Cerebral	30,93	8380,75	34,28	1870,00
		Traqueal	30,95	9906	ND	ND
7	*Pontoporia blainvillei*	Blowhole	30,92	8983,5	ND	ND
		Cerebral	31,69	2389,5	ND	ND
		Tracheal	32,23	3538,5	ND	ND
8	*Pontoporia blainvillei*	Oral	35,21	449	ND	ND
		Small intestine	33,56	1380	ND	ND
9	*Pontoporia blainvillei*	Blowhole	36	221,25	ND	ND
		Small intestine	36,2	220,001	ND	ND
10	*Pontoporia blainvillei*	Cerebral	34,5	717,5	ND	ND
		Large intestine	37	105,32	ND	ND
		Blowhole	34,92	445,5	ND	ND
11	*Pontoporia blainvillei*	Oral	34,16	704	ND	ND
12	*Pontoporia blainvillei*	Blowhole	33,16	1389,25	ND	ND
		Anal	35,45	294,75	ND	ND
14	*Pontoporia blainvillei*	Blowhole	34,8	459	ND	ND
16	*Pontoporia blainvillei*	Blowhole	33,94	600,75	ND	ND
17	*Pontoporia blainvillei*	Blowhole	33,12	1427,75	ND	ND
18	*Pontoporia blainvillei*	Tracheal	33,8	850,5	ND	ND
27	*Tursiops gephyreus*	Anal	33,06	2091	ND	ND
		Oral	32,51	1486	ND	ND
28	*Tursiops gephyreus*	Blowhole	37,17	92,003	ND	ND
		Anal	35,28	330,75	ND	ND
		Oral	37,04	105,35	ND	ND
34	*Steno bredanensis*	Oral	33,89	1095	ND	ND
35	*Steno bredanensis*	Blowhole	35,25	426,5	ND	ND
36	*Lagenodelphis hosei*	Blowhole	35.1	472,5		ND
37	*Kogia sima*	Blowhole	33,93	1618,5	ND	ND
		Ocular	35,83	2647,25	ND	ND
39	*Stenella clymene*	Mammary slit	28,95	33650000	37,1	500
		Blowhole	30,45	472500	ND	ND
